# Pannexin-1 channel “fuels” by releasing ATP from bone marrow cells a state of sterile inflammation required for optimal mobilization and homing of hematopoietic stem cells

**DOI:** 10.1007/s11302-020-09706-1

**Published:** 2020-06-12

**Authors:** Monika Cymer, Katarzyna Brzezniakiewicz-Janus, Kamila Bujko, Arjun Thapa, Janina Ratajczak, Krzysztof Anusz, Michał Tracz, Agnieszka Jackowska-Tracz, Mariusz Z. Ratajczak, Mateusz Adamiak

**Affiliations:** 1 Center for Preclinical Studies and Technology, Department of Regenerative Medicine, Medical University of Warsaw, ul. Żwirki i Wigury 61, 02-091 Warsaw, Poland; 2 Department of Hematology, Multi-specialist Hospital Gorzow Wlkp, University of Zielona Gora, Zielona Gora, Poland; 3 Stem Cell Institute at James Graham Brown Cancer Center, University of Louisville, Louisville, KY USA; 4 Institute of Veterinary Medicine, Department of Food Hygiene and Public Health Protection, Warsaw University of Life Sciences (WULS-SGGW), Warsaw, Poland

**Keywords:** Pannexin-1, Extracellular ATP, Nlrp3 purinergic signaling, Complement cascade, Stem cell mobilization, Stem cell homing, Stem cell engraftment

## Abstract

An efficient harvest of hematopoietic stem/progenitor cells (HSPCs) after pharmacological mobilization from the bone marrow (BM) into peripheral blood (PB) and subsequent proper homing and engraftment of these cells are crucial for clinical outcomes from hematopoietic transplants. Since extracellular adenosine triphosphate (eATP) plays an important role in both processes as an activator of sterile inflammation in the bone marrow microenvironment, we focused on the role of Pannexin-1 channel in the secretion of ATP to trigger both egress of HSPCs out of BM into PB as well as in reverse process that is their homing to BM niches after transplantation into myeloablated recipient. We employed a specific blocking peptide against Pannexin-1 channel and noticed decreased mobilization efficiency of HSPCs as well as other types of BM-residing stem cells including mesenchymal stroma cells (MSCs), endothelial progenitors (EPCs), and very small embryonic-like stem cells (VSELs). To explain better a role of Pannexin-1, we report that eATP activated Nlrp3 inflammasome in Gr-1^+^ and CD11b^+^ cells enriched for granulocytes and monocytes. This led to release of danger-associated molecular pattern molecules (DAMPs) and mitochondrial DNA (miDNA) that activate complement cascade (ComC) required for optimal egress of HSPCs from BM. On the other hand, Pannexin-1 channel blockage in transplant recipient mice leads to a defect in homing and engraftment of HSPCs. Based on this, Pannexin-1 channel as a source of eATP plays an important role in HSPCs trafficking.

**Electronic supplementary material:**

The online version of this article (10.1007/s11302-020-09706-1) contains supplementary material, which is available to authorized users.

## Introduction

Hematopoietic transplants are since more than 50 years the most successful therapeutic applications of stem cells. Initially, hematopoietic stem/progenitor cells (HSPCs) were harvested by multiple aspirations from donor bone marrow (BM). In subsequent years, two other important sources of HSPCs emerged that are mobilized peripheral blood (PB) and umbilical cord blood (UCB). With all these sources of cells, very often, a number of HSPCs that are present in harvested BM, mobilized PB, or UCB unit are limited [1–3]. In humans, a successful transplantation requires a sufficient number of harvested HSPCs per kg body weight of the patient, and a fast, consistent, and long-term multilineage engraftment requires the intravenous infusion of a minimum of 2 × 10^6^ CD34^+^ stem cells/kg recipient body weight; however, a higher dose of 5 × 10^6^ CD34^+^ cells/kg is considered preferable. Therefore, novel strategies are needed to increase a number of harvested HSPCs after mobilization as well as to enhance their “seeding efficiency” after transplantation into hematopoietic BM niches [3].

The procedure of HSPCs mobilization for transplant purposes is performed after administration of cytokine granulocyte colony-stimulating factor (G-CSF) or a small inhibitor of CXCR4 receptor, which besides blocking activity has some partial agonistic effect against CXCR4 [4–6]. As it is well known in autologous transplantation settings, ~ 10% of normal patients and ~ 25% of post-chemotherapy patients do not respond efficiently to currently recommended mobilization protocols and are deemed poor mobilizers. Therefore, there is a need to optimize mobilization procedures [7–9]. On the other hand, a low number of HSPCs present in mobilized PB, harvested BM, or single UCB unit require as high as possible their homing or seeding efficiency into BM niches after infusion to the patient recipient [10]. This is crucial to assure fast hematopoietic recovery, improve overall prognosis, and lower overall costs of transplant procedure.

Our group in the past years extensively investigated a role of innate immunity-induced BM sterile inflammation in HSPC mobilization and homing. Our recent intriguing data demonstrates that extracellular adenosine triphosphate (eATP) is a trigger of Nlrp3-inflammasome-induced sterile inflammation in BM microenvironment after administration of G-CSF and AMD3100. Moreover, eATP also induces a state of sterile inflammation in BM microenvironment after radio/chemotherapy myeloablative conditioning for transplant [11, 12].

eATP is released from innate immunity cells including granulocytes and monocytes, as well as from the other cells in BM microenvironment such as mesenchymal stroma cells and endothelial cells in response to pro-mobilizing drugs or myeloablative conditioning for transplant by radio/chemotherapy [11, 13–15]. This initiates a sequel of events that maintain a state of sterile inflammation in BM microenvironment. Since eATP is released from cells mainly by cell membrane expressed Pannexin-1 channel, we employed specific blocking peptides of Pannexin-1 to study its effect in mobilization and homing/engraftment of HSPCs [15–18].

Our data performed in murine model reveal a crucial role of Pannexin-1 channel in releasing eATP during pharmacological mobilization or myeloablative conditioning for transplantation and provides further evidence for a crucial role eATP plays in trafficking of HSPCs.

## Materials and methods

### Animals

Pathogen-free, 6–8-week-old C57BL/6 J (WT) mice were purchased from the Central Laboratory for Experimental Animals or Medical University of Warsaw or the Jackson Laboratory (Bar Harbor, ME, USA) at least 2 weeks prior to experiments. Animal studies were approved by the Animal Care and Use Committee of the University of Louisville (Louisville, KY, USA) and Warsaw Medical University (Warsaw, Poland).

### In vivo mobilization studies

Mice were mobilized with G-CSF (Amgen, Thousand Oaks, CA, USA) for 3 days at 100 μg/kg/day by subcutaneous injection (SC) or with AMD3100 (Sigma-Aldrich, St. Louis, MO, USA) for 1 day at 5 mg/kg by intraperitoneal injection (IP). Mobilized studied mice received also ^10^Panx (WRQAAFVDSY; 10 mg/kg for 10 consecutive days, intravenous injection (IV)). At 6 h after the last G-CSF injection, 1 h after AMD3100 injection, mice were bled from the retro-orbital plexus for plasma and hematology analysis, and PB was obtained from the vena cava (with a 25-gauge needle and 1-ml syringe containing 250 U heparin). MNCs were obtained by hypotonic lysis of RBCs in BD Pharm Lyse buffer (BD Biosciences) as described [19–24].

### Evaluation of hematopoietic stem/progenitor cell mobilization

For evaluation of circulating colony-forming unit-granulocyte/macrophage (CFU-GM) and SKL cells, the following formulas were used: (number of white blood cells [WBCs] × number of CFU-GM colonies)/number of WBCs plated = number of CFU-GM per ml of PB, and (number of WBCs × number of SKL cells)/number of gated WBCs = number of SKL cells per μl of PB [19–23].

### Peripheral blood parameter counts

To obtain white and red blood cell counts, 50 μl of PB was taken from the retro-orbital plexus of mice into microvette EDTA-coated tubes (Sarstedt Inc., Newton, NC, USA) and run on a HemaVet 950FS hematology analyzer (Drew Scientific Inc., Oxford, CT, USA) within 2 h of collection [19, 21].

### Clonogenic colony-forming unit-granulocyte/macrophage assay

Peripheral blood mononuclear cells (PBMNCs) were resuspended in human methylcellulose base medium (R&D Systems, Minneapolis, MN, USA), supplemented with 25 ng/ml recombinant murine granulocyte/macrophage colony-stimulating factor (mGM-CSF; PeproTech, Rocky Hill, NJ, USA) and 10 ng/ml recombinant murine interleukin 3 (mIL-3; PeproTech). Cells were incubated for 7 days (37°C, 95% humidity, and 5% CO_2_), and the numbers of CFU-GM colonies were scored using an inverted microscope (Olympus, Center Valley, PA, USA). Final results were recalculated based on the number of PBMNCs/μl of PB, as described above [25, 26].

### Fluorescence-activated cell sorting analysis

For staining of Lin^−^/Sca-1^+^/c-Kit^+^ (SKL cells), Lin^−^/Sca-1^+^/CD45^+^ (HSCs), Sca-1^+^/Lin^−^/CD45^−^ (VSELs), Lin^−^/CD45^−^/CD31^+^ (EPCs), and Lin^−^/CD45^−^/CD31^−^/CD90^+^ (MSCs), the following monoclonal antibodies were used: FITC–anti-CD117 (also known as c-Kit, clone 2B8; BioLegend, San Diego, CA, USA) and PE–Cy5–anti-mouse Ly-6 A/E (also known as Sca-1, clone D7; eBioscience, San Diego, CA, USA). All anti-mouse lineage marker antibodies, including anti-CD45R (also known as B220, clone RA3-6B2), anti-Ter-119 (clone TER-119), anti-CD11b (clone M1/70), anti-T cell receptor β (clone H57–597), anti-Gr-1 (clone RB6-8C5), anti-TCRγδ (clone GL3), and anti-CD45 (clone 30-F11), conjugated with PE; anti-CD31 (clone MEC 13.3), conjugated with APC; and anti-CD90.2 (clone 30-H12), conjugated with BV510, were purchased from BD Biosciences. Staining was performed in RPMI-1640 medium containing 2% FBS. All monoclonal antibodies were added at saturating concentrations, and the cells were incubated for 30 min on ice, washed twice, and analyzed with a FACSVerse cytometer (BD Biosciences) [26].

### Isolation of Gr-1+ cells

Gr-1+ cells were isolated from the BM of C57BL/6 mice. Briefly, BM was flushed from femurs, and the population of total nucleated cells was obtained after lysis of red blood cells (RBCs) using 1 × BD Pharm Lyse buffer (BD Pharmingen, San Jose, CA, USA). The cells were subsequently stained with phycoerythrin (PE)–anti-Gr-1 antibody (anti-Ly-6G and Ly-6C, clone RB6-8C5) for 30 min in medium containing 2% fetal bovine serum (FBS). The cells were then washed, resuspended in RPMI-1640 medium, and sorted using a Moflo XDP cell sorter (Beckman Coulter, Indianapolis, IN, USA) as populations of granulocytes (SSC^high^Gr-1+).

### qRT-PCR analysis of Nlrp3 inflammasome complex gene expression

Sorted Gr-1+ cells from C57BL/6 mice resuspended in RPMI-1640 medium plus 0.5% bovine serum albumin (BSA; Sigma-Aldrich) (2 million cells per 500 μl of medium) were incubated for 2 h at 37°C. Subsequently, cells were stimulated by adding G-CSF (100 ng/ml), ATP (250 ng/ml), S1P (0.01 μM), and AMD3100 (3 μM) or medium alone as control, and cells were incubated for 6 h at 37°C. Cells were centrifuged, and total RNA was isolated with the RNeasy Mini kit (Qiagen Inc.) after DNase I (Qiagen Inc.) treatment. The purified RNA was reverse-transcribed with MultiScribe Reverse Transcriptase, oligo(dT), and a random-hexamer primer mix (all from Applied Biosystems Life Technologies, CA, USA). Quantitative evaluation of the target genes was then performed using an ABI Prism 7500 sequence detection system (Applied Biosystems Life Technologies) with Power SYBR Green PCR Master Mix reagent and specific primers. The PCR cycling conditions were 95°C (15 s), 40 cycles at 95°C (15 s), and 60°C (1 min). According to melting point analysis, only one PCR product was amplified under these conditions. The relative quantity of a target gene, normalized to the β2-microglobulin gene as the endogenous control and relative to a calibrator, was expressed as 2^–ΔΔCt^ (fold difference).

The following primer pairs were used for analysis:

- mNLRP1
  - Forward primer: 5′- GCTGAATGACCTGGGTGATGGT-3′
  - Reverse primer: 5′-CTTGGTCACTGAGAGATGCCTG-3′
- mIL-18
  - Forward primer: 5′-ACAACTTTGGCCGACTTCAC-3′
  - Reverse primer: 5′-GGGTTCACTGGCACTTTGAT-3′
- mIL-1b
  - Forward primer: 5′-TCACAGCAGCACATCAACAA-3′
  - Reverse primer: 5′-TGTCCTCATCCTGGAAGGTC-3′
- mAIM2
  - Forward primer: 5′-AAAACTGCTCTGCTGCCTCT-3′
  - Reverse primer: 5′-GATGGCTTCCTGTTCTGCCA-3′
- mCasp1
  - Forward primer: 5′-CAC AGC TCT GGA GAT GGT GA-3′
  - Reverse primer: 5′-GGT CCC ACA TAT TCC CTC CT-3′
- mNlrp3
  - Forward primer: 5′-GCT GCT GAA GAT GAC GAG TG-3′
  - Reverse primer: 5′-TTT CTC GGG CGG GTA ATC TT-3′
- mHMGB1
  - Forward primer: 5′-TAA AAA GCC CAG AGG CAA AA-3′
  - Reverse primer: 5′-GCS GCS ATG GTC TTC CAC CT-3′
- mβ2M
  - Forward primer: 5′-ATGCTATCCAGAAAACCCCTCAAAT-3
  - Reverse primer: 5′-AACTGTGTTACGTAGCAGTTCAGTA-3′

### Evaluation of the mtDNA by real-time qPCR

Mice were injected once by G-CSF (100 μg/kg) (Amgen, Thousand Oaks, CA, USA). At 6 h after G-CSF injection, the mice were bled from the retro-orbital plexus for bone marrow analysis. Bone marrow cells were suspended in RPMI 1640 medium supplemented with l-glutamine and antibiotics (ThermoFisher Scientific). Then, the bone marrow cells were incubated in the culture medium for 1 h at 37 °C, after that they were centrifuged at 1800 r.p.m. (10 min). The cell supernatant and bone marrow cells were transferred to new tubes and frozen at − 20 °C until further analysis. Total DNA from bone marrow cells supernatant were isolated by using Genomic Mini Kit (A&A Biotechnology) and measured the concentration of DNA using NanoDrop. The resulting DNA fragments were amplified using the SYBR Green system (Bio-Rad). Primer sequences for the genes encoding 16S rRNA (mitochondrial), ND1 (mitochnodrial), and hexokinase 2 (HK2 nuclear encoded gene) are as follows:

- 16S rRNA
  - Forward primer: 5′-CCGCAAGGGAAAGATGAAAGAC-3′
  - Reverse primers: 5′-TCGTTTGGTTTCGGGGTTTC-3’
- ND1
  - Forward primer: 5′-CTAGCAGAAACAAACCGGGC-3′
  - Reverse primers: 5′-CCGGCTGCGTATTCTACGTT-3’
- HK2
  - Forward primer: 5′-GCCAGCCTCTCCTGATTTTAGTGT-3′
  - Reverse primer: 5′-GGGAACACAAAAGACCTCTTCTGG-3′

Analysis of mtDNA/nDNA ratio was calculated by using the following formula: ΔCt = Ct (nDNA gene) − Ct (mtDNA gene), copies of mtDNA = 2 × 2^ΔCt^, relative mtDNA content = mtDNA G-CSF treated mice/mtDNA control mice.

### Fluorescence-activated cell sorting analysis of apoptosis Gr-1+ cells from bone marrow

Mice were injected once by G-CSF (100 μg/kg) (Amgen, Thousand Oaks, CA, USA). At 1 h, 6 h, and 24 h after G-CSF injection, the mice were bled from the retro-orbital plexus for bone marrow analysis. For Gr-1+ cells from the bone marrow, the following antibodies were used: PE Annexin V, and 7-AAD (PE Annexin V Apoptosis Detection Kit I, BD Biosciences). All antibodies were added at saturating concentrations, and the cells were incubated for 15 min in the dark, suspended in 1× Annexin V Binding Buffer, and analyzed with a FACSVerse (BD Biosciences). Flow cytometer protocol: late apoptosis: 7-AAD (+), PE Annexin V (+); apoptosis: PE Annexin V (+).

### Enzyme-linked immunosorbent assay

Mice received ATP (3 days, 15 mg/kg, IP) or adenosine (3 days, 5 mg/kg, IP). Murine blood was obtained from the vena cava (1-ml syringe containing 100 μl of 0.5 M EDTA). Plasma samples were prepared by taking the top fraction after centrifugation at 600×*g* for 10 min at 4 °C and immediately freezing at − 80 °C. The residual C5a level was measured by enzyme-linked immunosorbent assay (ELISA) according to the manufacturer’s protocols (Abcam, cat. no. ab193718). Results are presented as % of control [21, 22].

### Short-term homing experiments

Mice received ^10^Panx (WRQAAFVDSY) or Scrambled 10Panx (^SC^Panx (FSVYWAQADR))—10 mg/kg for 10 consecutive days, intravenous injection (IV). One day before the last ^10^Panx or ^SC^Panx injection, mice were irradiated with a lethal dose of γ-irradiation (10Gy). Twenty-four hours later, animals were transplanted (by tail vein injection) with 5 × 10^6^ BM cells from WT mice labeled with PKH67 Green Fluorescent Cell Linker (Sigma-Aldrich, St Louis, MO, USA) according to the manufacturer’s protocol. At 24 h after transplantation, BM cells from the femurs were isolated via Ficoll-Paque and divided. A part of the cells was analyzed on a flow cytometer. The rest of the cells were plated in serum-free methylcellulose cultures and stimulated to grow CFU-GM colonies with granulocyte/macrophage colony-stimulating factor (GM-CSF, 25 ng/ml) and interleukin 3 (IL-3, 10 ng/ml). After 7 days of incubation (37°C, 95% humidity, and 5% CO_2_), the number of colonies was scored under an inverted microscope [27, 28].

### Evaluation of engraftment

Mice received ^10^Panx (WRQAAFVDSY) or Scrambled 10Panx (^SC^Panx (FSVYWAQADR))—10 mg/kg for 16 consecutive days, intravenous injection (IV). Eleven days before the last ^10^Panx or ^SC^Panx injection, mice were irradiated with a lethal dose of γ-irradiation (10Gy). Twenty-four hours after irradiation, mice were transplanted with 1.5 × 10^5^ BM cells from WT mice by tail vein injection. Twelve days after transplantation, femora of transplanted mice were flushed with phosphate-buffered saline (PBS). BM cells purified via Ficoll-Paque were plated in serum-free methylcellulose cultures and stimulated to grow CFU-GM colonies with G-CSF (25 ng/ml) and IL-3 (10 ng/ml). After 7 days of incubation (37°C, 95% humidity, and 5% CO_2_), the number of colonies was scored under an inverted microscope. Spleens were also removed, fixed in Telesyniczky’s solution for CFU-S assays, and colonies were counted on the surface of the spleen [27–29].

### Recovery of leukocytes and platelets

Mice received ^10^Panx (WRQAAFVDSY) or Scrambled 10Panx (^SC^Panx (FSVYWAQADR))—10 mg/kg every second day for 34 days, intravenous injection (IV). Twenty-six days before the last ^10^Panx or ^SC^Panx injection, mice were irradiated with a lethal dose of γ-irradiation (10Gy). Twenty-four hours later, mice were transplanted by tail-vein injection with 7.5 × 10^5^ BM cells from WT mice. Transplanted mice were bled at various intervals from the retro-orbital plexus to obtain samples for white blood cell (WBC) and platelet (PLT) counts as described [27, 28, 30]. Briefly, 50 μl of PB was taken into EDTA-coated Microvette tubes (Sarstedt Inc., Newton, NC, USA) and run within 2 h of collection on a HemaVet 950FS hematology analyzer (Drew Scientific Inc., Oxford, CT, USA).

### Statistical analysis

All results are presented as mean ± SD. All the results are presented as mean ± SD. Statistical analysis of the data was done using unpaired Student’s *t* test or one-way ANOVA followed by Dunnett’s multiple comparisons test (*p < 0.05).

## Results

### Inhibition of Pannexin-1 channel by specific blocking peptide ^10^Panx impairs pharmacological mobilization of stem cells from bone marrow into peripheral blood

In our previous work, we employed to block Pannexin-1 channel in mice a drug Probenecid that is employed in clinic to increase uric acid excretion in gout patients that in addition possess some additional effect as Pannexin-1 channel inhibitor, and we noticed impaired G-CSF-mediated mobilization [11]. Herein, we employed instead of Probenecid a specific inhibitor of Pannexin-1 channel that is small blocking peptide ^10^Panx and evaluated its effect on both G-CSF- and AMD3100-mediated mobilization of HSPCs.

**Fig. 1:**
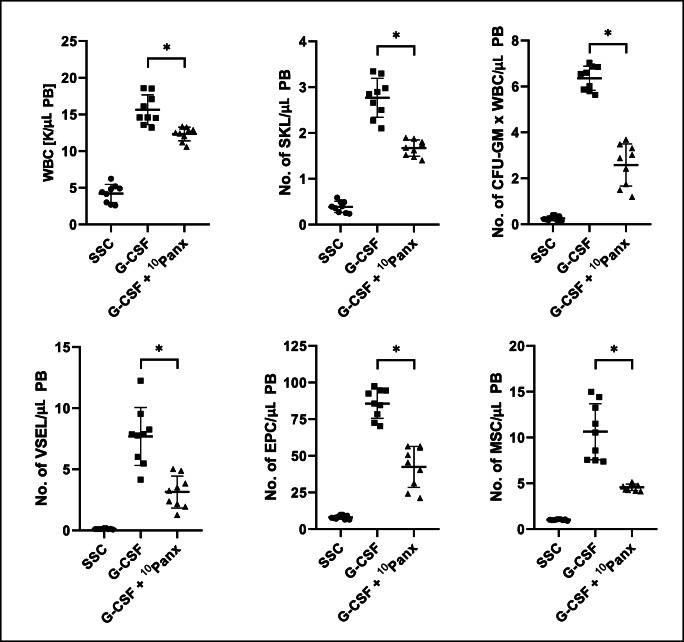
Blocking of Pannexin-1 channel decreases G-CSF mobilization of murine BM-residing stem cells. Mononuclear cells (MNCs) were isolated from WT mice after 3 days of G-CSF mobilization and treatment groups received additionally ^10^Panx blocking peptide. The numbers of WBCs, SKL (Sca-1^+^/c-kit^+^/Lin^−^) cells, CFU-GM clonogenic progenitors were evaluated in PB. SSC indicates non-mobilized mice under steady-state conditions. Results from two independent experiments are pooled together. *p < 0.05; comparing mobilized WT with mobilized WT administered with ^10^Panx

**Fig. 2:**
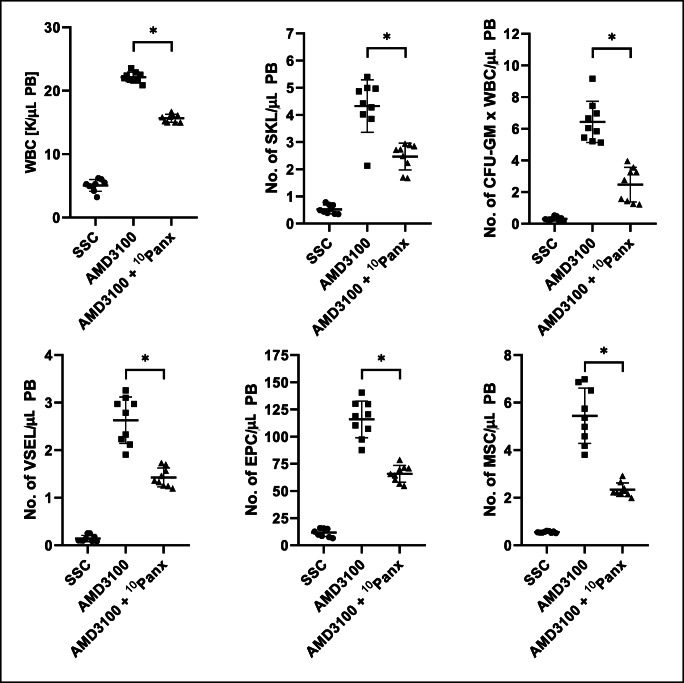
Blocking of Pannexin-1 channel decreases AMD3100 mobilization of murine BM-residing stem cells. Mononuclear cells (MNCs) were isolated from WT mice 1 h after 1 dose of AMD3100 mobilization and treatment groups received additionally Panx^10^ blocking peptide. The numbers of VSELs (Sca-1^+^/CD45^−^/Lin^−^), EPCs (CD45^−^/CD31^+^/Lin^−^) and MSCs (CD45^−^/CD31^−^/CD90^+^/Lin^−^) were evaluated in PB. SSC indicates non-mobilized mice under steady-state conditions. Results from two independent experiments are pooled together. *p < 0.05; comparing mobilized WT with mobilized WT administered with ^10^Panx

Supplementary Fig. 1 shows that ^10^Panx is not toxic against clonogeneic hematopoietic progenitor cells at doses employed in our studies. We noticed that blockage of Pannexin-1 channel by ^10^Panx affected release of HSPCs from BM into PB (Fig. 1) as well as also other types of BM-residing stem/progenitor cells including mesenchymal stroma cells (MSCs), endothelial progenitor cells (EPCs), and very small embryonic-like stem cells (VSELs) (Fig. 2).

Thus, data presented in Figs. 1 and 2 provide evidence that Pannexin-1 channel is involved by releasing eATP in mobilization of several types of stem/progenitor cells residing in BM niches. This also further confirms a role of Pannexin-1 channel in stress response to extracellular stimuli.

### Pannexin-1-released eATP activates Nlrp3 inflammasome, releases mitochondrial DNA, induces leucocyte pyroptosis, and triggers complement cascade activation

Nlrp3 inflammasome is one of the major sensors of changes in body microenvironments, cell activation, and cell metabolic activity. Initially, it has been reported to be expressed in innate immunity cells, but recent evidence indicates that it is expressed in several other types of cells including lymphocytes, HSPCs, MSCs, and bone marrow endothelium. eATP is a major mediator of purinergic signaling released from innate immunity cells after administration of pro-mobilizing drugs, which subsequently in autocrine/paracrine manner activates Nlrp3 inflammasome [11, 13, 14, 31, 32].

**Fig. 3:**
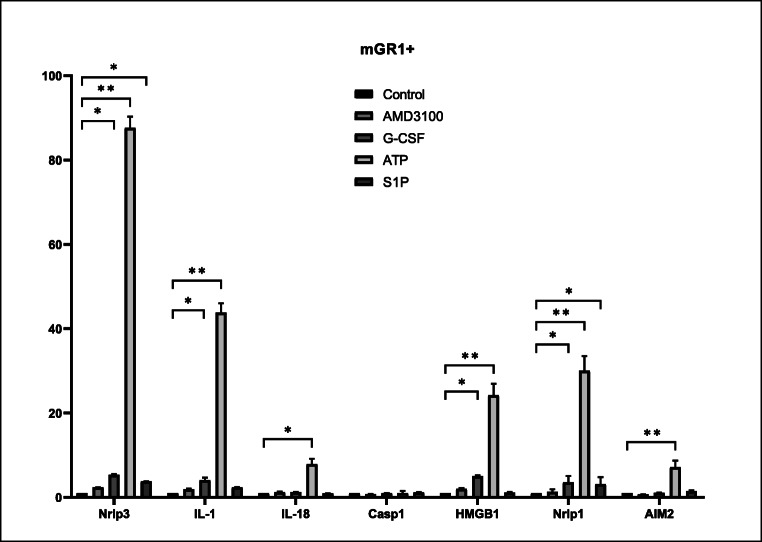
Expression of mRNA for the Nlrp3 inflammasome complex in mGr1^+^BM cells after stimulation with AMD3100, G-CSF, ATP and S1P measured by qRT-PCR. Expression of Nlrp1, Nlrp3, Aim-2, Hmgb1, IL-1β, Il-18, Casp1, mRNAs in murine bone marrow Gr-1^+^cells after stimulation for 6 h, in RPMI medium plus 0.5% BSA with AMD3100, G-CSF, ATP and S1P as measured by qRT-PCR. Results of qRT-PCR were normalized to the β2 microglobulin (β2m) level. Combined data from three independent experiments is shown. *p < 0.05; **p < 0.001

Figure 3 confirms that eATP as compared to G-CSF and AMD3100 increases high expression of Nlrp3 inflammasome components (Nlrp3, IL-1β and IL-18) and sorted by fluorescence-activated cell sorting (FACS) Gr-1^+^ granulocytes and CD11b^+^ monocytes belonging to innate immunity cells. In contrast, the level of caspase-1 mRNA seems to be unchanged. What is important is that our data also indicates that in addition to Nlrp3 mRNA also mRNA for Nlrp1 and Aim2 become upregulated after stimulation by eATP. Moreover, eATP in addition to inflammasome mRNA also strongly stimulated the expression of mRNA for high molecular group box-1 (HMGB1), an important intranuclear protein that is one of danger associated molecular pattern (DAMP) molecules or alarmines released from stressed or damaged cells [33].

**Fig. 4:**
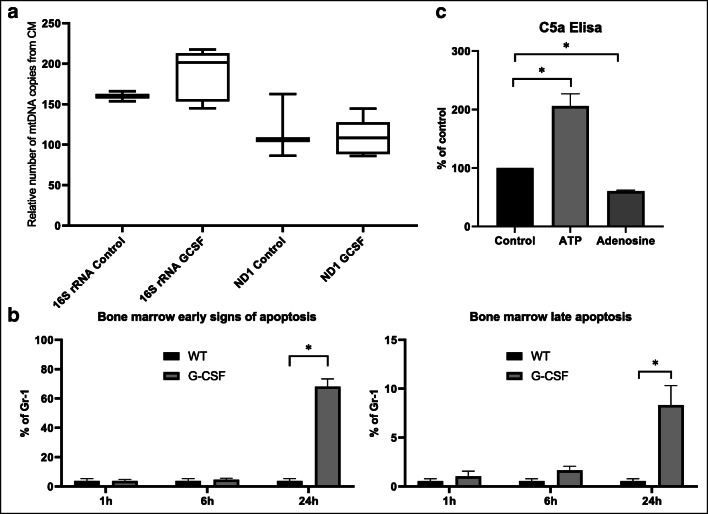
Release of mitochondrial DNA (mtDNA), induction of pyroptosis in Gr-1^+^ BMMNC and ComC activation. **a** Relative quantification was performed on control (WT) and G-CSF (100 μg/kg) treated samples in triplicates using qPCR by amplification of ND1 and 16S genes belonging to stable part of mtDNA, and normalized against hexokinase gene (HK). The increase in mtDNA number of copies in cell supernatant after G-CSF mobilization. Data are represented as box-and-whisker plot. Statistical differences were calculated using Student’s *t* test, *p < 0.001. **b** Bone marrow cells were stained using antibodies PE Annexin V, and 7-AAD (PE Annexin V Apoptosis Detection Kit I, BD Biosciences) and measured by flow cytometer. Results from flow cytometry are presented as a percentage of Gr-1+ cells from bone marrow. Statistically significant increase in early signs of early apoptosis and late apoptosis is observed after 24 h after G-CSF mobilization. Statistical differences were calculated using Student’s *t* test, *p < 0.001. **c** The level C5a protein in mouse plasma after treatment with ATP and adenosine measured by ELISA. Results are presented as a percentage of control. The data represent the mean value ± SEM for two independent experiments. *p < 0.05

It is known that extensive eATP signaling may lead to cell damage in mechanism of pyroptosis. In fact, as demonstrated in Fig. 4a, G-CSF-induced mobilization led to an increase of mitochondrial DNA in conditioned medium harvested from BM cells, which correlates with hyperactivation of Nlrp3 inflammasome inside cells that may damage integrity of mitochondria. This was paralleled by evidence of presence of early signs of apoptosis in BM Gr-1^+^ granulocytes by FACS 24 h after G-CSF administration (Fig. 4b left panel). We also detected that a significant percentage of BM Gr-1^+^ cells (~ 8%) displayed by FACS 24 h after administration of G-CSF signs of late apoptosis.

As expected, release of mitochondrial DNA and other DAMPs potentiates activation of complement cascade (ComC) in BM that is responsible for final execution of HSPCs release from BM. In fact, Fig. 4c shows that ComC became activated in response to eATP as indicated by an increase of plasma level of a cleavage fragment of activated fifth component of ComC (C5a).

### Bone marrow microenvironment-expressed Pannexin-1 is involved in homing and engraftment of transplanted hematopoietic stem/progenitor cells

Evidence accumulate that myeloablative conditioning for transplantation by irradiation or chemotherapy induces in BM microenvironment a state of “sterile inflammation” in which release of eATP plays an important role [14, 34, 35]. To address how important Pannexin-1 channel is in release of eATP for homing and engraftment of HSPCs, we performed BM transplants of BMMNC into mice exposed to Panx^10^ blocking peptide before transplantation.

**Fig. 5:**
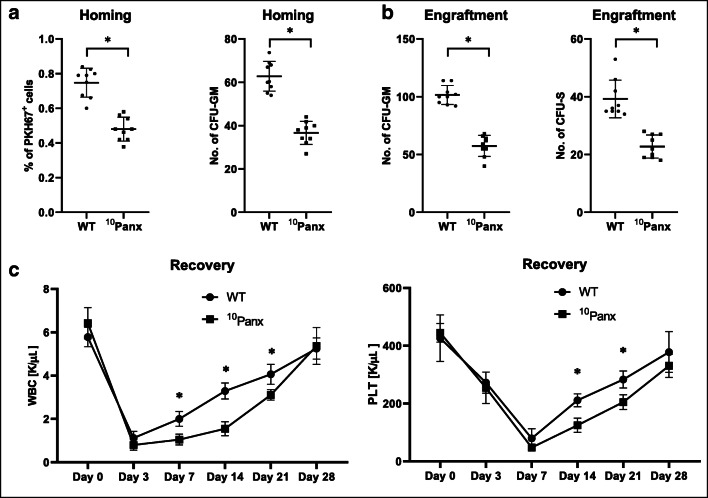
Impact of Pannexin-1 channel blocking on homing, short- and long-term engraftment of WT HSPCs. **a** Lethally irradiated mice (9 per group) treated with ^10^Panx were transplanted with bone marrow mononuclear cells (BMMNCs) from WT mice, labeled with a PKH67 cell linker. Twenty-four hours after transplantation, femoral BMMNCs were harvested, the number of PKH67 cells was evaluated by FACS, and the clonogenic CFU-GM progenitors were enumerated in an in vitro colony assay. **b** Lethally irradiated mice (9 per group) treated with ^10^Panx were transplanted with bone marrow mononuclear cells (BMMNCs) from WT mice, and 12 days after transplantation, spleens were removed for counting the number of CFU-S colonies and femoral BMMNCs were harvested and plating to count the number of CFU-GM colonies. No colonies were formed in lethally irradiated or untransplanted mice (irradiation control). *p < 0.05. **c** Lethally irradiated mice (9 per group) treated with ^10^Panx were transplanted with bone marrow mononuclear cells (BMMNCs) from WT mice. White blood cells and platelets were counted at intervals (at 0, 3, 7, 14, 21, and 28 days after transplantation). *p < 0.05. Data is shown combined from two independent experiments

Figure 5 a shows that the number of fluorochrome labeled donor cells 24 h after transplantation into recipient mice pretreated by ^10^Panx was reduced by 60% as compared to control animals. Similarly, the number of donor-derived CFU-GM in BM was reduced by more than 50% in recipient mice exposed to ^10^Panx. Next, we noticed that 12 days after transplantation, the number of donor-derived CFU-S in spleen as well as CFU-GM in BM was reduced in recipient mice pretreated by ^10^Panx by more than 50% after transplantation as compared to control animals (Fig. 5b). Corresponding with these results, we noticed that recipient mice pretreated by ^10^Panx and transplanted with BMMNC have impaired kinetic recovery of leukocytes and platelets (Fig. 5c). Supplementary Fig. 2 shows that in control experiments, Pannexin-1 scrambled peptide did not affect hematopoietic recovery of transplanted animals.

## Discussion

Hematopoiesis is regulated by several peptide-based cytokines, hormones and growth factors, bioactive lipids, steroid hormones and that is highly relevant to our current work by extracellular nucleotides and purinergic signaling [36–39]. The most important mediator of purinergic signaling is extracellular adenosine triphosphate (eATP) that activates a family of P2 purinergic receptors [15]. eATP is released from activated, stressed or damaged cells by involving different secretory mechanisms. The most important is release of eATP through Pannexin-1 that belong to family of cell membrane expressed large transmembrane channels connecting the intracellular and extracellular space. These channels allow the passage of ions and small molecules between these compartments including eATP [15, 40]. Recent evidence indicates that in addition to Pannexin-1 channels also connexin 43 that usually forms gap junctions between neighboring cells may release eATP if it is has access to extracellular space [41–43]. Finally, eATP could also be released by extracellular microvesicles [44, 45]. Nevertheless, Pannexin-1 seems to be the most important path for eATP release.

It is well known that eATP activates in P2X7 and P2X4 receptor-dependent manner Nlrp3 inflammasome, which is a multiprotein oligomer complex and important component of the innate immunity network expressed by myeloid cells, including Gr-1^+^ granulocytes, CD11b^+^ monocytes, and dendritic cells [46]. The Nlrp3 inflammasome in response to eATP activates intracellular caspase 1. Activation of this enzyme promotes maturation and secretion of pro-inflammatory interleukin 1β (IL-1β) and interleukin 18 (IL-18). This process depends on caspase 1-mediated proteolytic cleavage of the cytokine pro-forms (pro-IL-1β and pro-IL-18). Mature cleaved IL-1β and IL-18 are subsequently released into the extracellular space [46]. All this together maintains a state of sterile inflammation in BM microenvironment.

In our previous work, we demonstrated that eATP and purinergic signaling involving activation of P2X7 receptor and Nlrp3 inflammasome plays an important role in pharmacological mobilization of HSPCs as well as other types of BM-residing stem cells into PB [11]. We have also demonstrated that if we employed an unspecific Pannex-1 channel inhibitor that is Probenecid, a drug employed in the clinic to lower blood level of uric acid to treat gout and gouty arthritis, the G-CSF-induced mobilization of HSPCs was significantly impaired [11]. Herein, to get a better insight into this phenomenon, we employed ^10^Panx, a short Pannexin-1 channel blocking peptide and extended our mobilization studies for not only G-CSF but also AMD3100-induced mobilization. In parallel, we investigated if exposure of lethally irradiated recipient mice to Pannexin-1 channel blocking peptide will affect homing and engraftment of intravenously injected BM cells.

We noticed that exposure of mice before mobilization to Pannexin-1 blocking peptide, but not to control scrambled ones, inhibits both G-CSF, and AMD3100-induced mobilization of HSPCs as well as other types of BM-residing stem cells such as MSCs, EPCs and VSELs. We also compared efficacy of eATP activation of inflammasome components in a population belonging to innate immunity Gr-1^+^ granulocytes and CD11b^+^ monocytes and noticed that eATP as compared to G-CSF, AMD3100, and S1P is the most potent activator on Nlrp3 inflammasome elements in these cells. We postulate that the first wave of eATP release leading to Nlrp3 inflammasome activation is triggered after administration by G-CSF or AMD3100, and later on eATP in an autocrine/paracrine manner that sustains inflammasome activation in innate immunity cells.

Sustained activation of Nlrp3 inflammasome during mobilization induces release of ROS and mitochondrial DNA from eATP-activated cells that together with other released danger-associated molecular pattern (DAMP) molecules such as HMGB1 or S1008A/9 proteins activate complement cascade (ComC) that as we demonstrated in the past is crucial for egress of HSPCs from BM into PB [22, 47]. Moreover, it has been reported that G-CSF administration induces apoptosis in osteoblasts lining trabecular bones in BM [48]. We demonstrate herein that administration of G-CSF induced apoptotic changes in BM-residing Gr-1^+^ and CD11b^+^, and we explain this by increase in eATP release that induces pyroptosis in these cells. Interestingly, while we observed by FACS analysis early signs of apoptosis in innate immunity cells 24 h after administration of G-CSF, we did not notice it after administration of AMD3100. This indicates that there are still different mechanisms mediating mobilization of HSPCs in response to G-CSF vs. AMD3100.

Next, based on our previous observation that lethal irradiation of mice leads to release of eATP from BM [11], and our data that eATP secreted from BM of myeloblated for transplantation recipients may play a role in homing and engraftment of HSPCs [28], to better assess a role of eATP, we exposed mice to be transplanted to Panexin-1 inhibitor.

Accordingly, a crucial role in homing and engraftment of HSPCs in BM after transplantation is played by α-chemokine stromal-derived factor-1 (SDF-1), which chemoattracts CXCR4^+^ HSPCs to BM niches [49–52]. However, the fact that CXCR4^−/−^ HSPCs still home and engraft at a significant level in the BM of normal wild-type animals [53] prompted us to investigate other homing factors besides SDF-1 that may support the homing function of the SDF-1–CXCR4 axis. To address the contribution of two other chemoattractants (and major homing factors) for HSPCs, namely, sphingosine-1-phosphate (S1P) and eATP, we transplanted HSPCs from CXCR4^fl/fl^ Cre^Tg/−^ mice, in which CXCR4 had been eliminated by a Cre hematopoietic driver strategy, into sphingosine kinase-1-deficient (Sphk1^−/−^) mice, which are deficient for S1P expression in BM [28]. These experiments revealed a compensatory role for other homing factors that support the SDF-1–CXCR4 and S1P–S1P_1_R homing axes. Since ATP is also a chemoattractant for HSPCs, we proposed that eATP compensates for their deficiency. Therefore, to get better evidence for involvement of eATP in homing/engraftment of HSPCs, we exposed transplant recipient mice to Pannexin-1 blocking peptide and report herein for the first time that blockage of Pannexin-1 channel in murine recipient BM impairs both homing and engraftment of HSPCs.

We postulate that eATP level in conditioned transplantation BM provides chemotactic gradient for HSPCs that supports major BM homing factor that is SDF-1, but also we cannot exclude a possibility that eATP induces some homing and engraftment facilitating changes in BM microenvironment. This however requires further studies to investigate what molecular changes are induced by eATP and purinergic signaling in hematopoietic microenvironment in myeloablated host, and we are currently performing such experiments.

In conclusion, we provide further evidence for a role of Pannexin-1 in eATP release-mediated induction of sterile inflammation in BM tissue required for optimal mobilization of HSPCs. We also demonstrate for a first time that Pannexin-1 plays a role in eATP-mediated homing and engraftment of HSPCs after transplantation into myeloablated recipient. This data generated in murine models shed more light on eATP and purinergic signaling-mediated trafficking of HSPCs.

## Electronic supplementary material

Fig. 1. Toxicity studies of 10Panx (WRQAAFVDSY) were performed based on evaluation for the number of CFU-GM and BFU-E clonogenic progenitors in in vitro assays. Murine bone marrow mononuclear cells were incubated with medium alone or different doses of 10Panx for 1h and then supplemented for CFU-GM and BFU-E colonies. BM hematopoietic clonogeneic progenitors were scored after 7 days of incubation, data from two separate experiments are pooled together. (PPTX 49 kb)

Fig. 2. Panel A. Lethally irradiated mice treated with Scrambled 10Panx (SCPanx (FSVYWAQADR)) were transplanted with bone marrow mononuclear cells (BMMNCs) from WT mice, labeled with a PKH67 cell linker. 24 hours after transplantation, femoral BMMNCs were harvested, the number of PKH67 cells was evaluated by FACS, and the clonogenic CFU-GM progenitors were enumerated in an in vitro colony assay. Panel B. Lethally irradiated mice (9 per group) treated with SCPanx were transplanted with bone marrow mononuclear cells (BMMNCs) from WT mice, and 12 days after transplantation spleens were removed for counting the number of CFU-S colonies and femoral BMMNCs were harvested and plating to count the number of CFU-GM colonies. *p < 0.05. Lethally irradiated mice (9 per group) treated with SCPanx were transplanted with bone marrow mononuclear cells (BMMNCs) from WT mice. (PPTX 80 kb)
