# Supplementary figures and images for: Pannexin-1 channel “fuels” by releasing ATP from bone marrow cells a state of sterile inflammation required for optimal mobilization and homing of hematopoietic stem cells
Source: Purinergic Signal. 2020 Jun 12;16(3):313–25. doi: 10.1007/s11302-020-09706-1 (PMC7524928; doi:10.1007/s11302-020-09706-1)

## Slide 1
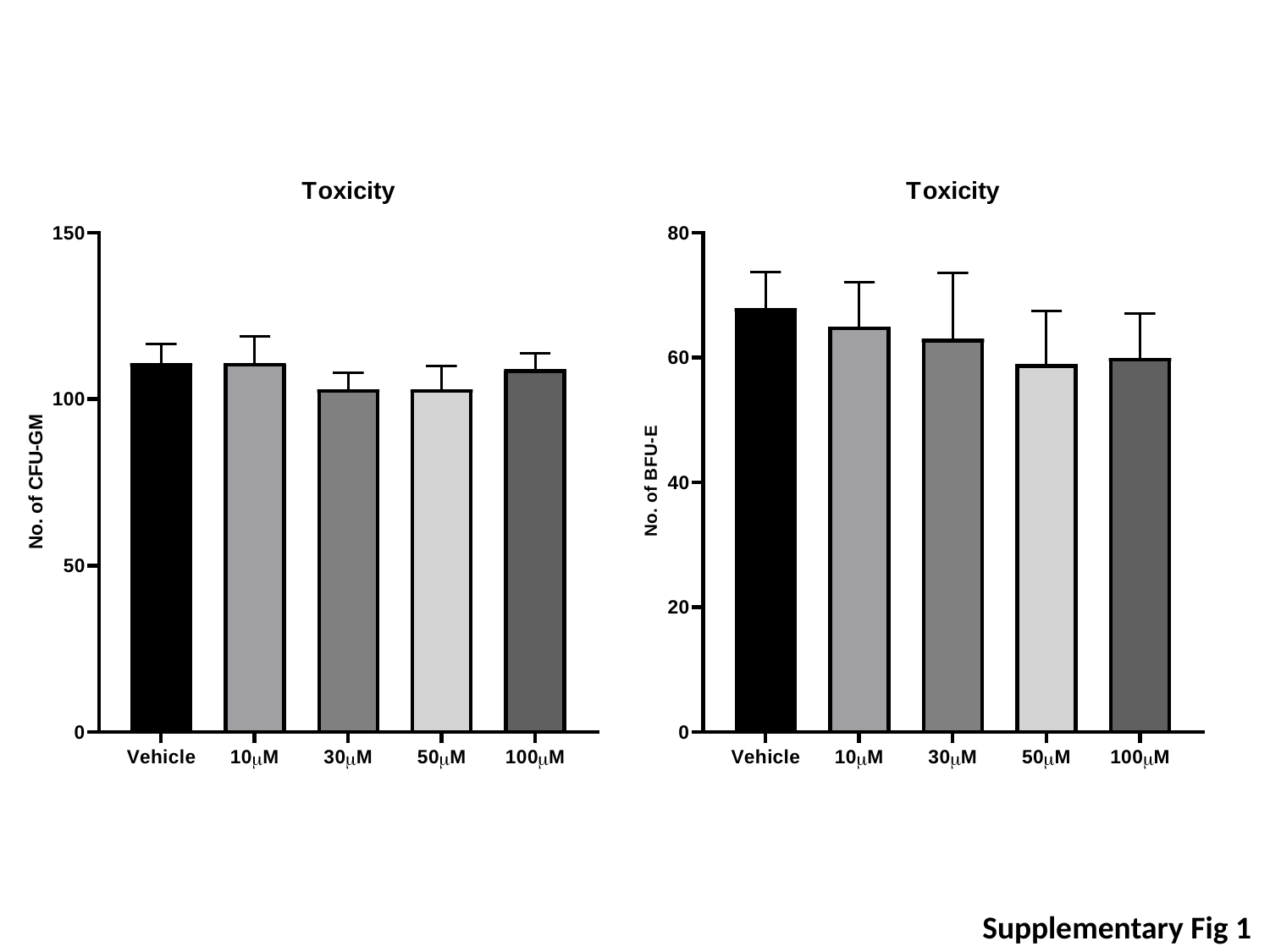

Supplementary Fig 1

Supplement: Supplementary file 1 — Toxicity studies of 10Panx (WRQAAFVDSY) were performed based on evaluation for the number of CFU-GM and BFU-E clonogenic progenitors in in vitro assays. Murine bone marrow mononuclear cells were incubated with medium alone or different doses of 10Panx for 1h and then supplemented for CFU-GM and BFU-E colonies. BM hematopoietic clonogeneic progenitors were scored after 7 days of incubation, data from two separate experiments are pooled together. (PPTX 49 kb) [file 11302_2020_9706_MOESM1_ESM.pptx]

## Slide 1
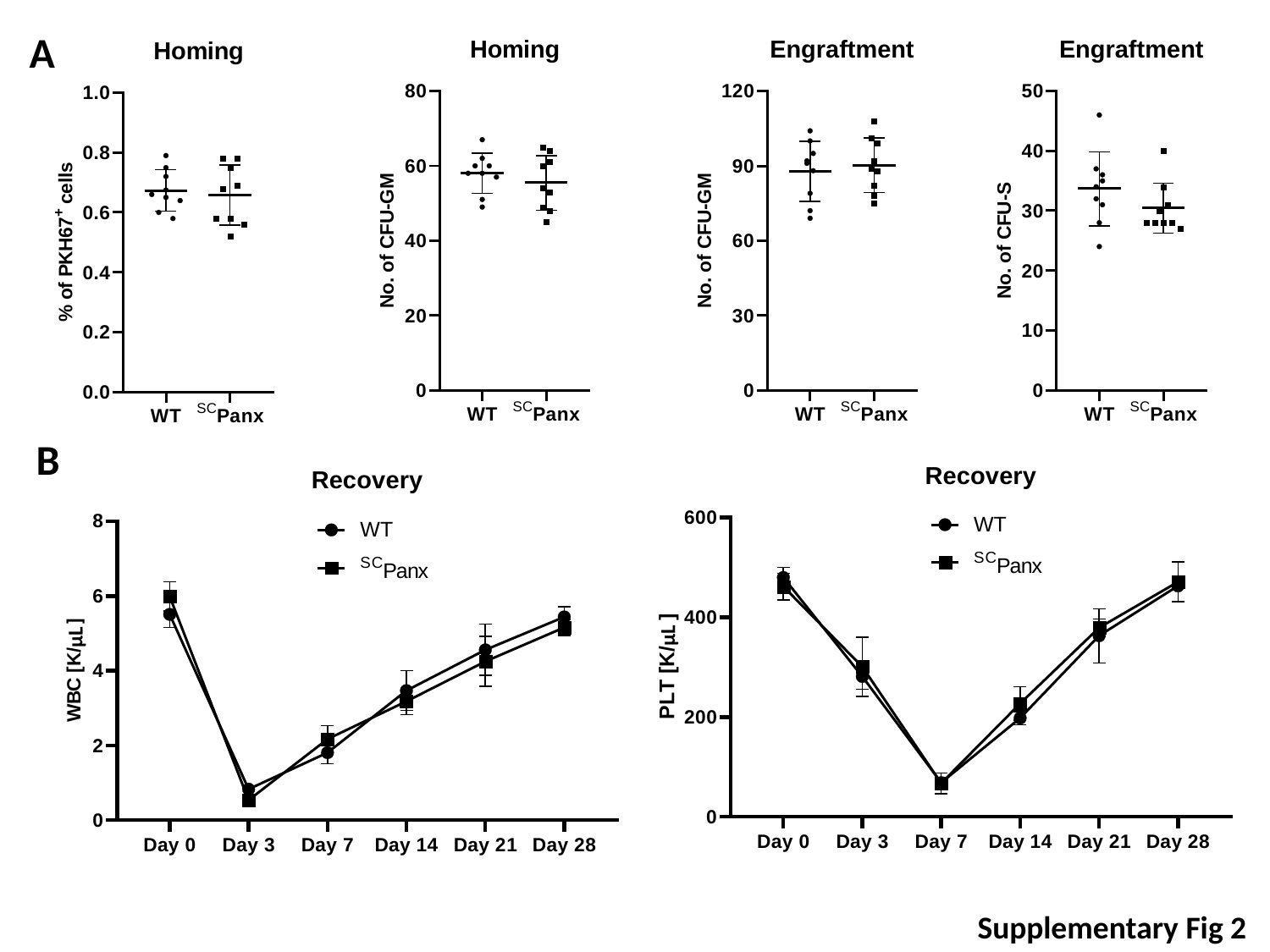

A
B
µL
µL
Supplementary Fig 2

Supplement: Supplementary file 2 — Panel A. Lethally irradiated mice treated with Scrambled 10Panx (SCPanx (FSVYWAQADR)) were transplanted with bone marrow mononuclear cells (BMMNCs) from WT mice, labeled with a PKH67 cell linker. 24 hours after transplantation, femoral BMMNCs were harvested, the number of PKH67 cells was evaluated by FACS, and the clonogenic CFU-GM progenitors were enumerated in an in vitro colony assay. Panel B. Lethally irradiated mice (9 per group) treated with SCPanx were transplanted with bone marrow mononuclear cells (BMMNCs) from WT mice, and 12 days after transplantation spleens were removed for counting the number of CFU-S colonies and femoral BMMNCs were harvested and plating to count the number of CFU-GM colonies. *p < 0.05. Lethally irradiated mice (9 per group) treated with SCPanx were transplanted with bone marrow mononuclear cells (BMMNCs) from WT mice. (PPTX 80 kb) [file 11302_2020_9706_MOESM2_ESM.pptx]
